# Flexible bioelectronic innovation for personalized health management

**DOI:** 10.1002/cai2.61

**Published:** 2023-03-12

**Authors:** Maowen Xie, Guang Yao, Yuan Lin

**Affiliations:** ^1^ School of Materials and Energy University of Electronic Science and Technology of China Chengdu Sichuan China; ^2^ State Key Laboratory of Electronic Thin Films and Integrated Devices University of Electronic Science and Technology of China Chengdu Sichuan China; ^3^ Medico‐Engineering Cooperation on Applied Medicine Research Center University of Electronic Science and Technology of China Chengdu Sichuan China; ^4^ Shenzhen Institute for Advanced Study University of Electronic Science and Technology of China Shenzhen Guangdong China

**Keywords:** bioelectronics, flexible, innovation

## Abstract

With the vigorous development of intelligent medical care and interdisciplinary science, innovative flexible bioelectronics (FBEs) are emerging in health monitoring, disease diagnosis and treatment, and even cancer therapy. This work comments on the recent progress of FBEs in personalized health management, emphasizing its innovative role in cancer therapy. Future perspectives on the challenges and opportunities for the next‐generation innovative FBEs are also proposed.

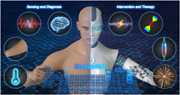

**Abbrevations**
FBE: flexible bioelectronics
NIR: near‐infrared
PDT: photodynamic therapy
PTT: photothermal therapy

## 1 INTRODUCTION

With the rapid developments in intelligent medical care and interdisciplinary science, innovative flexible bioelectronics (FBEs) are emerging for monitoring health, diagnosing and treating diseases, and even cancer therapy. FBEs subvert physical form and break through the bottleneck of traditional rigid electronics, rendering intimate and non‐invasive contact with the human body to stably capture physiological signals or accurately administer therapeutic interventions. Like other biomedical devices and systems, miniaturization, multi‐functionalization, and intelligence are current challenges and directions for future developments in FBEs. Future perspectives on the challenges and opportunities for the next generation of innovative FBEs are also proposed.

## 2 INNOVATIVE FLEXIBLE BIOELECTRONICS (FBEs) FOR SIGNAL SENSING AND DISEASE DIAGNOSTICS

**Figure 1:**
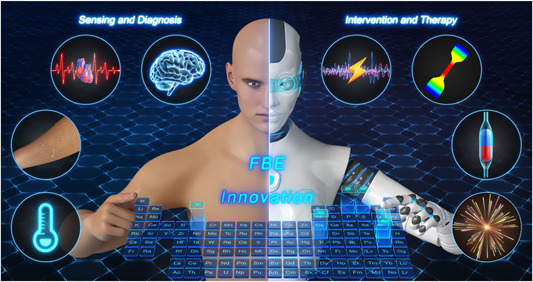
Innovative flexible bioelectronics for personalized health management.

Innovative FBEs are portable electronic devices that integrate sensors into or combine with the human body to collect and manage personal health records using mobile devices. Most existing wearable FBEs focus on monitoring biophysical or biochemical parameters such as body movement, respiration rate, heart rate, oxygen saturation, sweat volume, and calories [1], which are critical primary data for health management (Figure 1). For example, the FBE‐based tactile sensor (capacitive, resistive, optical, electromagnetic, piezoelectric), which has three‐dimensional force perception capability to adjust grasping posture and grasping force in real time through a tactile perception feedback function, can accurately identify the grasped object. These FBEs have great application value for intelligent prosthetics, medical rehabilitation, and surgical robots [2]. Bodily fluids such as blood, tears, sweat, urine, and saliva contain physiologically relevant molecules and biomarkers that can provide helpful information for medical diagnostics. FBEs can monitor biomarker‐rich bodily fluids non‐invasively to provide insights into patient's health at the molecular level. Additionally, levels of electrolytes such as Na^+^, K^+^, NH^4+^, and Ca^2+^, and pH and metabolites such as glucose, uric acid, lactate, ascorbic acid, and urea can be analyzed by biosensors, providing valuable information for healthcare suggestions [3]. For instance, physiological and psychological stress can be dynamically assessed by analyzing concentrations of cortisol, glucose, and vitamin C in sweat. However, a single sensing signal cannot fulfill the current needs for monitoring health. Thus, multifunctional co‐sensing FBEs have become the mainstream of flexible health electronics research. FBEs integrate different material systems and functional units onto flexible substrates [4], such as ultrasound transducers and electrochemical sensors that simultaneously monitor blood pressure, heart rate, lactate, and caffeine in sweat. In general, FBEs for signal sensing and disease diagnosis are combined with the human body in the form of tattoos, gloves, clothes, dental braces, armbands, electronic skin, and flexible contact lenses to record and calculate data on various biochemical markers in human bodily fluids and mechanical movements of the body.

Accurate health assessments require real‐time monitoring of multiple physical and biochemical signals; however, simultaneously capturing and calibrating numerous signals remains a considerable challenge. Ensuring that the materials are nontoxic to humans is the foremost step. The long‐term effects of these functional materials in humans are still uncertain and require further long‐term studies in animals/humans for validation. It is also difficult for FBEs to operate stably in complex environments. The organic reagents commonly used to monitor biomarkers are easily contaminated, inactivated, or degraded, making it challenging to maintain stable performance in a dynamic environment. To achieve biochemical and biophysical hybrid sensing functions, various material‐based complex units need to be integrated into a limited area, which inevitably increases the stiffness of FBEs. Additionally, due to the complexity of multiplexed signals, on‐site signal processing circuitry and sensor calibration mechanisms for accurate analysis of the physiological state need to be improved.

## 3 INNOVATIVE FBEs FOR DISEASE INTERVENTION AND TREATMENT

Surgery and medication are standard approaches to treating diseases. However, several rounds of surgery are associated with high cost, discomfort, and time commitments. Recently, nonpharmacological optical, electrical, and mechanical interventions from innovative FBEs have been extensively applied in disease treatment (Figure 1). Photothermal therapy (PTT), photodynamic therapy (PDT), photobiomodulation (PBM), and optogenetic therapy are widely used in emerging photonic healthcare applications [5]. An optical machine vision system for motion capture technology is also essential for rehabilitation robots and intelligent prosthetics [6]. Similarly, electrical stimulation technology has been increasingly investigated in the fields of transcranial electrical stimulation, transcorneal electrical stimulation, and spinal cord stimulation in the form of earplugs, headbands, and contact lenses. FBEs for transdermal diagnosis and treatment have improved skin compatibility and multifunctionality. In particular, iontophoretic transdermal drug delivery systems that accelerate transdermal penetration and extraction by applying a continuous low‐voltage current have already been commercialized for rapid drug delivery [7]. In summary, FBEs primarily utilize nonpharmacological photon therapy, current stimulation, and mechanical motion for disease intervention and treatment in wound repair, hair growth, neuromodulation, targeted drug release, gesture recognition, and athletic rehabilitation.

Noninvasive FBEs have significant advantages for disease treatment but still have a long way to go before clinical application. First, their robustness and effectiveness should be further improved to ensure that they work appropriately under strict intervention conditions (including specific light, magnetic field, electric field, and temperature) and at adequate intervention depths. Second, FBEs should be adequately designed and encapsulated to avoid side effects such as itching, burning, and stinging. Third, timely feedback on the degree of disease is also required, and correlations between disease stages and different intervention modes need to be clarified.

## 4 INNOVATIVE FBEs FOR CANCER THERAPY

Cancer is the leading cause of death worldwide and has a relatively insidious course. Patients usually have related symptoms in midterm or terminal disease, making cancer treatment difficult and expensive. Early screening of tumor markers is critical for cancer prevention and treatment. Currently, clinical detection of cancer primarily relies on imaging techniques and genomic or proteomic detection. These techniques are time‐consuming and require expensive, sophisticated instruments.

Optical methods have played an essential role in detecting cancer biomarkers by inducing photochemical and biological reactions to activate photosensitive materials and biological tissues. PTT uses the photothermal effect induced by photothermal agents to increase the temperature of surrounding tissue and trigger cell death, while also inevitably causing thermal damage to normal tissues. PDT is used to irradiate tumor sites with specific wavelengths, activate photosensitive drugs in tumor tissues to trigger photochemical reactions, and generate high‐activity monomorphic oxygen (^1^O_2_) to oxidize nearby biological macromolecules, producing cytotoxic effects that kill tumor cells. A hypoxic microenvironment can promote tumor growth and metastasis, and adequate oxygen supply is a prerequisite to ensure the effectiveness of PDT. PBM devices use red or near‐infrared (NIR) light to heal and repair damage caused by injury or disease, such as brain damage, nerve regeneration, wound healing, and hair growth stimulation. In particular, PBM has beneficial effects for managing soft tissue necrosis and oral mucositis in patients with head and neck cancer; there are also potential applications for treating xerostomia, dysgeusia, radiodermatitis, and breast cancer‐related lymphedema [5, 8, 9, 10].

In the early stages of cancer, only trace biomarkers exist. Hence, the reliability of the diagnostic tests and the sensitivity of FBEs are crucial. Electrochemical methods combined with novel nanomaterials and electroactive compounds provide higher sensitivity and low detection limits for novel biomarkers. Amperometry and impedimetry have been applied to detect cancer biomarkers for target binding and specific reactions in redox changes, such as breast biomarkers (carbohydrate antigen 15‐3, epidermal growth factor receptor, mucin‐1, and osteopontin), liver biomarkers (mir‐100‐5p, mir‐122, a‐Fetoprotein, HCCR‐1), prostate biomarkers (glutathione S‐transferase P1 gene, miR‐103a, miR‐106a, miR‐107), and human epithelial‐derived tumor biomarkers (folic acid protein). Due to the signal response generated near the sensor‐electrode surface, the activity of these biosensors is highly dependent on electrode characteristics [11, 12].

For cancer therapy, it is challenging to achieve the desired effects with single interventions. Many therapies require targeted drugs or multi‐physical synergy to achieve the optimal therapeutic effect and minimal toxic side effects; for example, chemotherapy, PTT, and PDT can effectively kill tumor cells at small drug dosage. Such multimodal combination therapies can induce tumor cell apoptosis and achieve better therapeutic effects by controlling drug delivery and release. A shrinkable nanomicelle complex FBE that dissociates under NIR laser irradiation after reaching the tumor could convert light energy into heat energy, realizing photothermal ablation of superficial tumor tissues. Concurrently, chemotherapeutic drugs are released and enter the tumor to for photothermal‐chemotherapy drug combination therapy for cancer [13]. The magnetocaloric phenomenon under an alternating electromagnetic field has been used to melt capsules and release certain drugs on demand [14]. In conclusion, electrochemical methods can efficiently detect trace cancer biomarkers in the early stages of cancer due to their advantages of high sensitivity and low detection limit. Optical methods using the photothermal effect to kill tumor cells or activate photosensitive drugs in tumor tissues to achieve controlled release have potential application value for tumor therapy. Additionally, multimodal combination therapy that integrates diagnosis and treatment generally has a better therapeutic effect [15].

## 5 CONCLUSION

This paper comments on the applications of FBEs for personalized health monitoring, disease intervention, and cancer therapy. Although significant breakthroughs have been made in FBEs, there continue to be challenges and problems. For example, FBEs for health monitoring still require innovations in functional materials, multi‐channel signal processing, biocompatibility, and material stability. In disease intervention, current challenges remain in device flexibility, synergetic intervention integration, and intervention parameter optimization. In particular, FBEs are increasingly being used for the early screening and treatment of cancer, realizing the integration of diagnosis‐intervention‐drug release to prevent disease progression and occurrence. These developments have laid a solid foundation for FBEs in intelligent medical care and have accelerated the construction of innovative healthcare platforms.

## AUTHOR CONTRIBUTIONS

**Maowen Xie:** Conceptualization (equal); investigation (equal). **Guang Yao:** Conceptualization (equal); formal analysis (equal); funding acquisition (equal); investigation (equal); methodology (equal). **Yuan Lin:** Formal analysis (equal); funding acquisition (equal).

## CONFLICT OF INTEREST STATEMENT

The authors declare no conflicts of interest.

## ETHICS STATEMENT

Not applicable.

## INFORMED CONSENT

Not applicable.

## Data Availability

Data sharing is not applicable to this article as no datasets were generated or analyzed during the current study. This comment did not generate new unique datasets.
